# Diamond sensors for hard X-ray energy and position resolving measurements at the European XFEL

**DOI:** 10.1107/S1600577524006015

**Published:** 2024-07-30

**Authors:** Tuba Çonka Yıldız, Wolfgang Freund, Jia Liu, Matthias Schreck, Dmitry Khakhulin, Hazem Yousef, Christopher Milne, Jan Grünert

**Affiliations:** a European XFEL, Holzkoppel 4, 22869Schenefeld, Germany; b Institut für Physik, Universität Augsburg, Universitätsstrasse 1, 86159Augsburg, Germany; Paul Scherrer Institut, Switzerland

**Keywords:** diamond sensor, diamond detector, scCVD diamond, electronic-grade diamond, photon diagnostics, X-ray free-electron lasers, pulse resolved

## Abstract

The diagnostics of X-ray beam properties has a critical importance at the European X-ray Free-Electron Laser facility. Besides existing diagnostic components, utilization of a diamond sensor was proposed to achieve radiation-hard, non-invasive beam position and pulse energy measurements for hard X-rays. In particular, with very hard X-rays, diamond-based sensors become a useful complement to gas-based devices which lose sensitivity due to significantly reduced gas cross-sections. The measurements presented in this work were performed with diamond sensors consisting of an electronic-grade single-crystal chemical-vapor-deposition diamond with position-sensitive resistive electrodes in a duo-lateral configuration. The results show that the diamond sensor delivers pulse-resolved X-ray beam position data at 2.25 MHz with an uncertainty of less than 1% of the beam size. To our knowledge this is the first demonstration of pulse-resolved position measurements at the MHz rate using a transmissive diamond sensor at a free-electron laser facility. It can therefore be a valuable tool for X-ray free-electron lasers, especially for high-repetition-rate machines, enabling applications such as beam-based alignment and intra-pulse-train position feedback.

## Abstract 2

Diamond sensors have been developed and tested for operation with high-energy X-ray beams at the European XFEL.

## 1. Introduction

The European X-ray Free-Electron Laser (EuXFEL) generates extremely intense X-ray pulses characterized by durations in the femtosecond range and high repetition rates up to 4.5 MHz (Decking *et al.*, 2020 ▸). Each pulse can contain several millijoules of energy within a pulse that is less than 100 fs long. These pulses are created via a process called self-amplified spontaneous emission (SASE) which has a stochastic nature causing shot-to-shot variations in beam intensity, spectrum and pointing angle. Therefore, it is important to perform single-shot beam energy and position measurements. Due to the fact that pulses need to be transmitted through the beamline to the user experiments, such measurements should be performed in a least-invasive manner, during which almost no beam loss occurs and beam properties do not change considerably. Detailed information on EuXFEL beam diagnostics is given by Grünert *et al.* (2019 ▸).

At the EuXFEL, X-ray gas monitors (XGMs) (Maltezopoulos *et al.*, 2019 ▸) are used as non-invasive intensity monitors. They are efficient in delivering pulse-resolved and absolutely calibrated pulse energy signals. The beam position can also be derived, but only averaged over several tens of seconds. Gas-based systems become less efficient when the photon energy increases towards harder X-rays (Maltezopoulos *et al.*, 2024 ▸) and at about 20 keV the measurement uncertainty of these systems under standard operating conditions becomes too large. Besides, it is not always possible to implement such systems requiring gas exchange system and differential pumping sections. X-ray imaging systems (Koch *et al.*, 2019 ▸) provide accurate beam profile and position information, but only at a low frame rate of 10 Hz. There is also a so-called backscatter monitor (Tono *et al.*, 2011 ▸) that detects X-rays backscattered from thin foils. However, the scattering cross section depends on the energy and with these monitors there is a problem of having a good calibration over the energy spectrum. To overcome these issues, sensors have been developed based on single-crystal diamonds produced by chemical-vapor-deposition (CVD). Diamond is a promising material not only for electronics and quantum devices but also for harsh radiation environments. In particular, a single-crystal diamond has superior characteristics for high-temperature, high-frequency, high-radiation and high-voltage applications. Synchrotron facilities have already applied diamond sensors as beam monitors (Pomorski *et al.*, 2009 ▸; Desjardins *et al.*, 2018 ▸; Morse *et al.*, 2010 ▸). The application at a free-electron laser (FEL) is even more challenging because a synchrotron delivers of the order of 10^12^ photons s^−1^ while FELs deliver a similar number of photons in each pulse. The FEL peak power can damage many materials in the beam path. As the pulse length is extremely short, there is hardly a chance for the removal of heat from the sensor material. Another factor is the high repetition rate of the FEL pulses in the MHz range, where the time between pulses is also too short to dissipate absorbed energy significantly. Thanks to all the superior properties of diamond, it is possible to use diamond sensors for the extremely bright and short pulses of X-ray free-electron lasers (XFELs) (Roth *et al.*, 2018 ▸).

With the motivation of achieving non-invasive position and energy measurements at 4.5 MHz for hard X-rays, the X-ray Photon Diagnostics (XPD) group of EuXFEL initiated an R&D project for the development of suitable diamond sensors. In particular, for very hard X-rays the gas-based devices lose sensitivity and diamond-based sensors are a useful complement.

## 2. Sensor properties

An electronic-grade single-crystal CVD (scCVD) diamond is the starting material for the detector. This kind of diamond is a promising detector material providing high charge collection efficiency, high mobility of charge carriers, high spatial and energy resolution, lower saturation field, radiation hardness, low energy and angular dependence. High-quality scCVDs (with area around 4 mm × 4 mm) produced by different companies, mostly from Element Six Ltd (https://www.e6.com), were purchased and polished down to a thickness of 20–50 µm. For the first detector that was mounted, both surfaces of the diamond were coated with a resistive diamond-like carbon (DLC) layer by pulsed laser deposition from a graphite target (Pomorski *et al.*, 2009 ▸). DLC has the advantage of having a very good adhesion to diamond and providing an only carbon composition within the detection area of the sensor. The low-*Z* sensor material has a low X-ray absorption coefficient, resulting in low absorbed power densities. In further detectors, surface graphitization by carbon implantation was used instead of DLC coating to create the resistive layer. The implantation of carbon in polished scCVD diamonds was performed at the University of Augsburg using an ion implanter (AXCELIS, formerly EATON) with a terminal voltage of 200 kV. For the signal collection and bias voltage supply, aluminium strip electrodes are deposited by sputter coating on the surface of the diamond, close to the edges, not touching the beam. Both implantation and sputtering were performed using custom-designed masks produced by EuXFEL.

**Figure 1:**
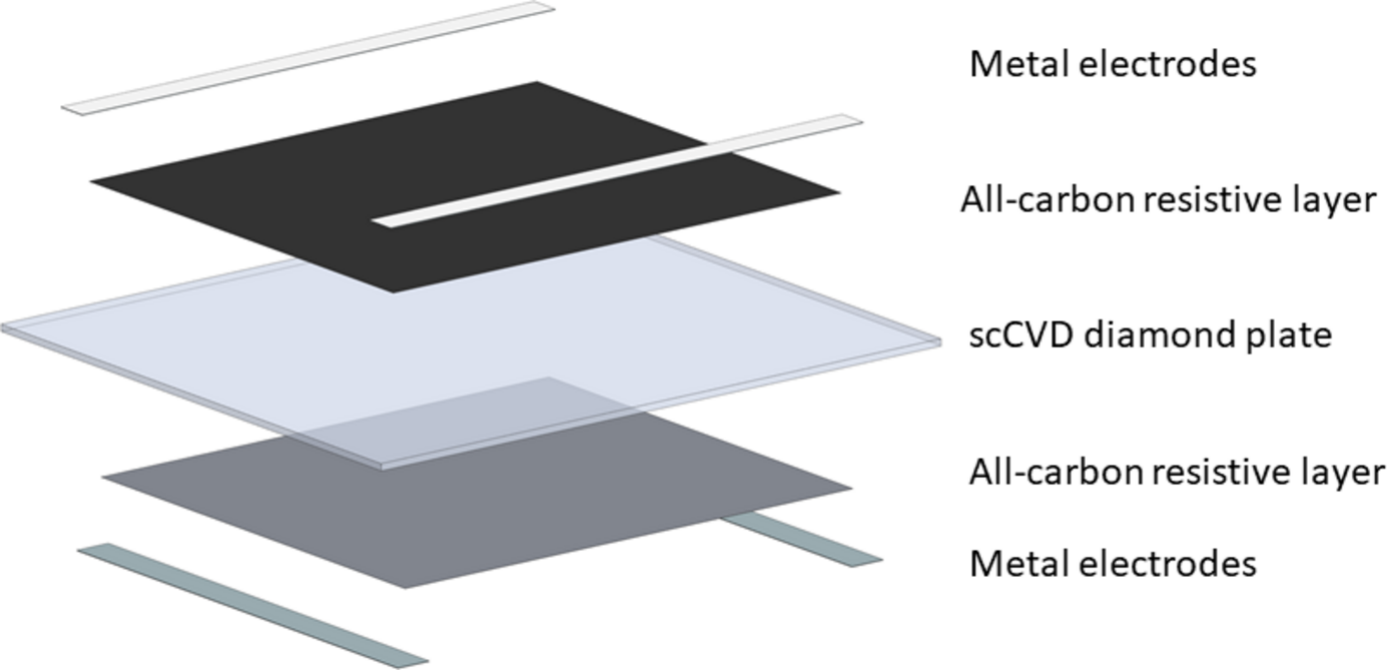
Exploded sketch of the structure of the diamond sensor. The all-carbon electrode is either a DLC layer or a graphitic layer formed by ion implantation and annealing.

The structure of the sensor is shown in Fig. 1 ▸. In the case of implantation, only the all-carbon resistive layer differs in the structure, such that the DLC layer is replaced by a layer of highly conductive graphitized diamond. This layer is formed by ion-implantation of carbon ions with energies of 70 keV and 170 keV, doses of 1 × 10^16^ cm^2^ and 2 × 10^16^ cm^2^, respectively. Since the defect profiles of 70 keV and 170 keV peak at different depths, the double implantation facilitates a more homogeneous amorphization profile. Appropriate energies and doses have been derived from SRIM (stopping and range of ions in matter) simulations. The subsequent annealing is performed in argon atmosphere for 15 min at 950°C. The depth of the amorphized diamond is approximately 0.23 µm (ignoring any swelling effects). A sheet resistance of 50 Ω/□ has been achieved with this kind of ion implantation, which improves the efficiency for the charge collection on the surface of the diamond. In comparison, applying the deposition process of DLC layers, the sheet resistance can be tuned between 1 kΩ/□ and 10 MΩ/□ as reported by Pomorski *et al.* (2009 ▸). On one hand, a low resistance guarantees a fast extraction of the generated charge out of the detector crystal before the arrival of the next pulse. On the other hand, position sensitivity decreases when the resistance becomes very low. The sheet resistance of 50 Ω/□ is a compromise between both requirements, taking into account the minimum period of 220 ns between successive pulses at the European XFEL. We would also like to highlight the excellent mechanical stability of the contact layers generated by ion implantation as proven by scratching tests with metal tweezers.

**Figure 2:**
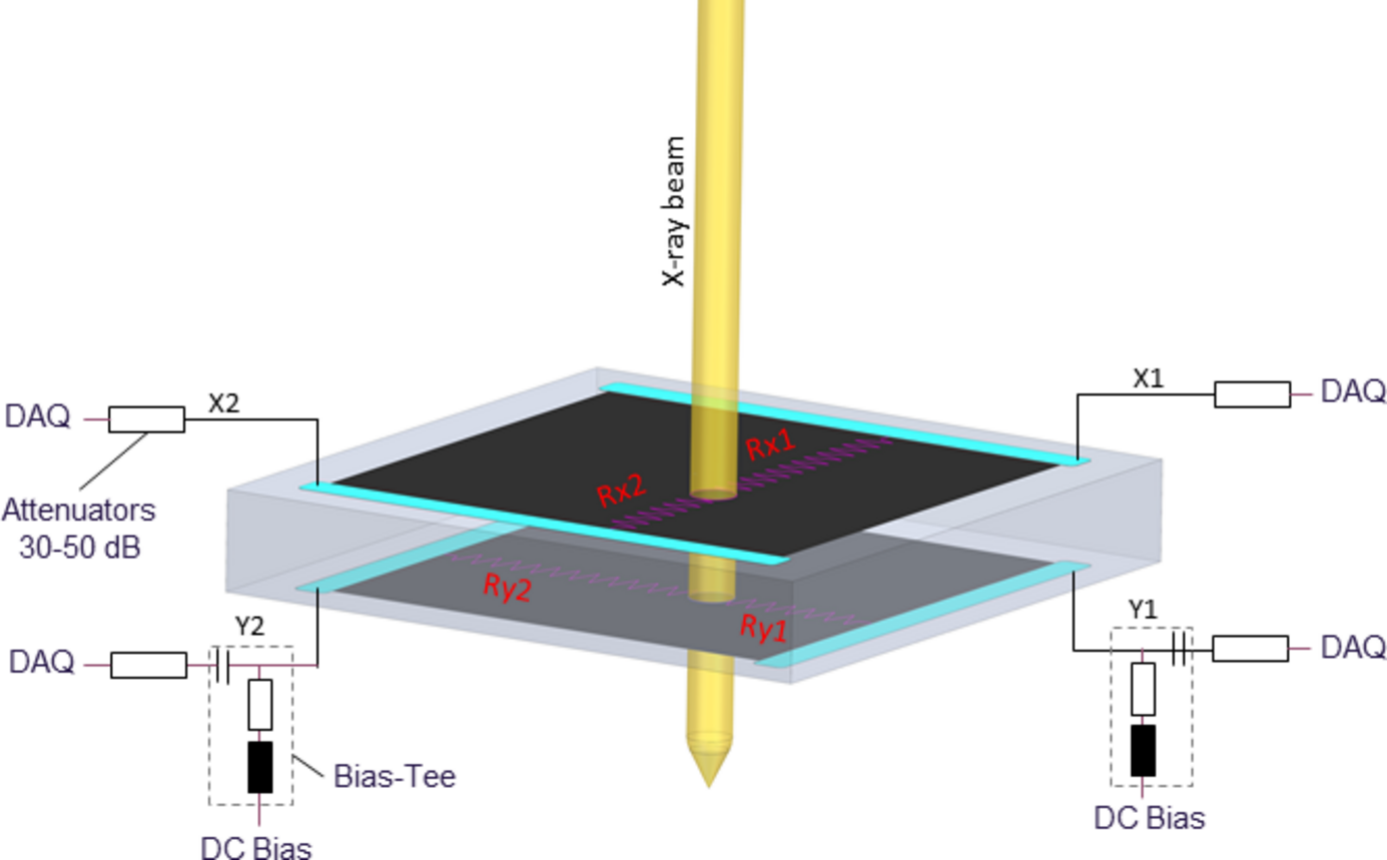
The working principle and the detailed structure of the duo-lateral diamond sensor.

A duo-lateral configuration was chosen for the electrodes in order to achieve position sensitivity in two dimensions (2D). In this configuration the two electrode pairs are perpendicularly positioned on each of the two surfaces. Alternatively, the electrodes may also be in the form of pixels, strips or quadrants on the surface of the diamond (Bloomer *et al.*, 2022 ▸; Bohon *et al.*, 2010 ▸). However, these alternatives are not appropriate for the FEL due to the highly coherent nature of the FEL radiation, which would experience wavefront distortion and interferences in the case of any non-homogeneity at the contacts. Besides, the SASE FEL beam has significant variations in pulse energy and pointing angle. A non-Gaussian beam with large deviations from the center trajectory would cause an unreliable measurement with a conventional quadrant detector. The principle of operation for a duo-lateral position-sensitive diamond sensor can be seen in Fig. 2 ▸ (Desjardins *et al.*, 2018 ▸).

As the X-ray beam passes through the diamond, electron–hole pairs are created. The electrodes on the surface are biased externally so that the charge carriers drift to the corresponding electrode. The charge collected at each electrode is transmitted to the readout electronics where a digital signal is created and processed further. To achieve this, the diamond is mounted on a custom-designed carrier board made of Al_2_O_3_ ceramic, which is fully UHV compatible. The electrical connection of the conducting signal cable to the electrodes is made so that the required low contact resistance is achieved and mechanical strain is avoided. As a result, all four channels are read out, and the *x*- and *y*-coordinate of the point of interaction can be obtained as shown in equation (1) using a center-of-gravity method (Pomorski *et al.*, 2009 ▸),
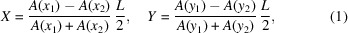
where *A*(*x*_*i*_) and *A*(*y*_*i*_) symbolize amplitudes, usually the amplitude of the X-ray induced charge collected on the lateral electrodes *x*_*i*_ and *y*_*i*_, respectively, and *L* corresponds to the distance between the lateral metal electrodes, which is approximately 3.2 mm.

**Figure 3:**
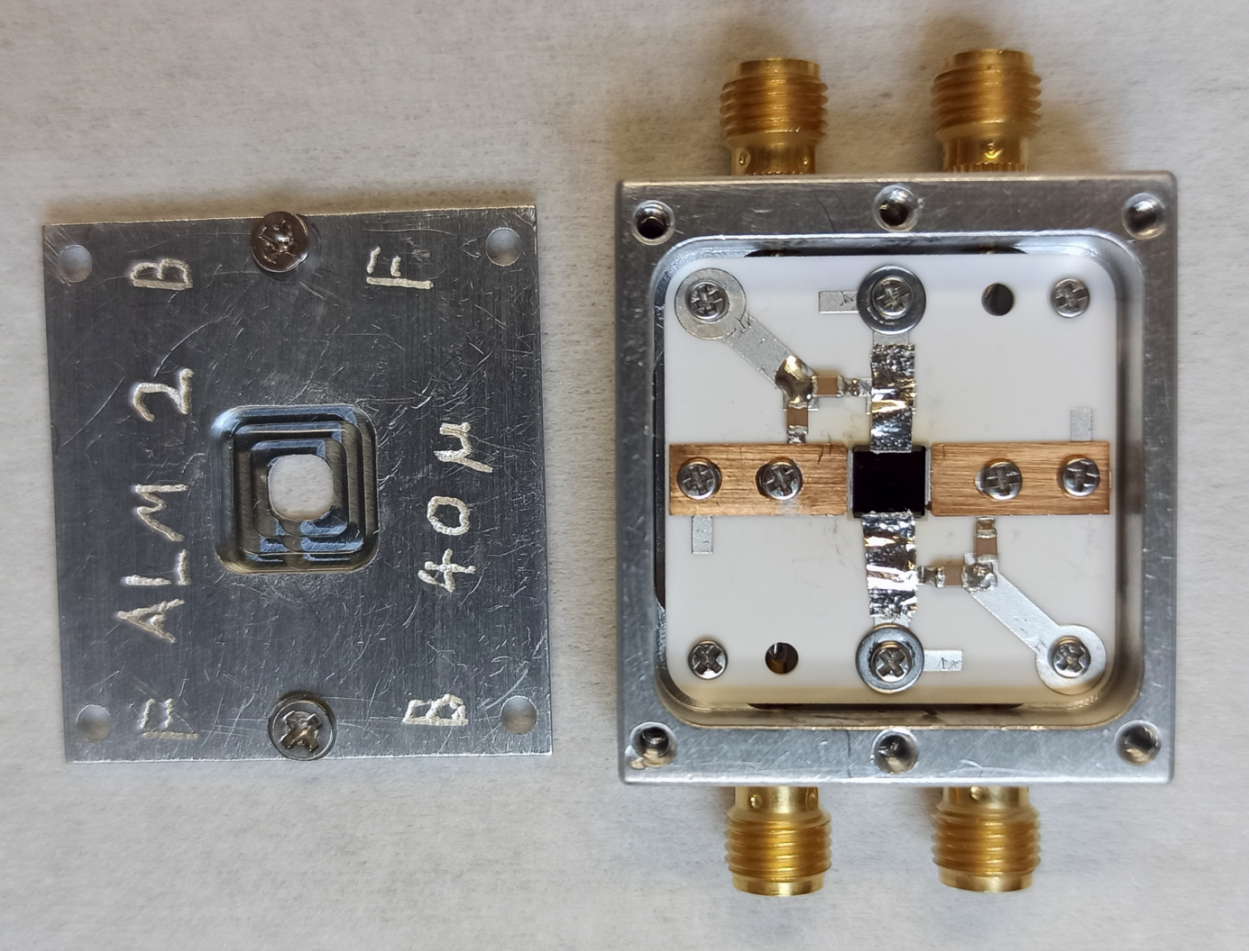
One of the diamond sensors mounted on a custom-designed ceramic carrier board in a metal housing.

The final view of the mounted diamond sensor can be seen in Fig. 3 ▸

## 3. Experimental setups

### 3.1. Experimental setup in the photon tunnel

**Figure 4:**
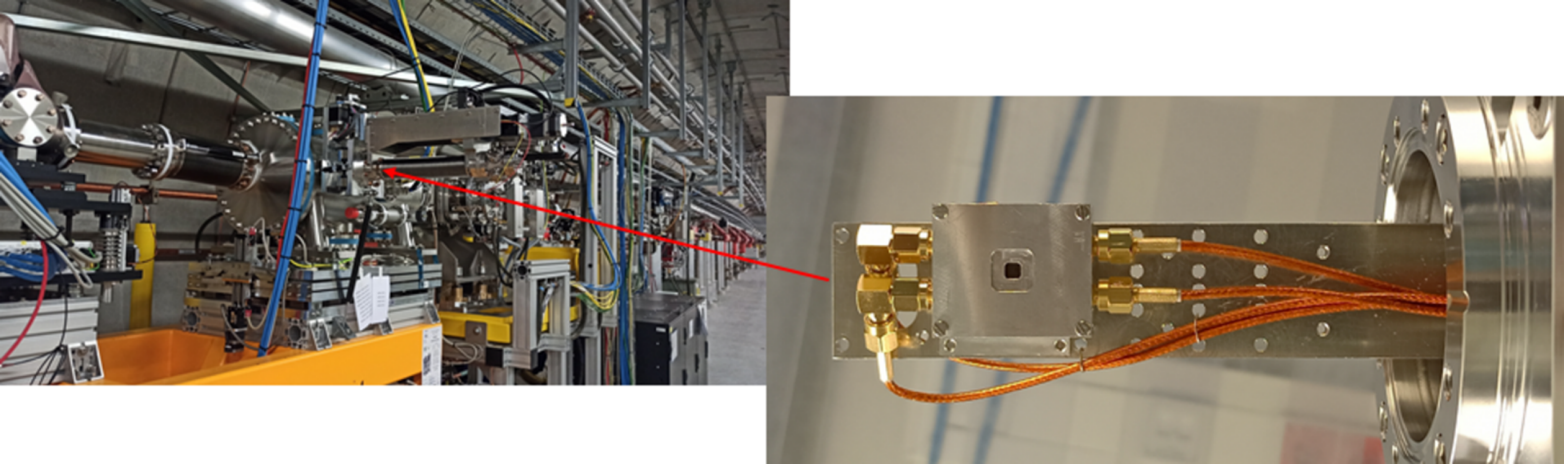
Right: diamond detector assembly mounted on an XY-manipulator (here the in-vacuum parts are shown). Left: placement in the SASE2 beamline tunnel XTD1.

The first measurements were performed using a DLC-coated sensor having a structure as shown in Fig. 1 ▸ with dimensions 4 mm × 4 mm and a thickness of 40 µm. It was mounted on an XY-motorized manipulator and attached to a K-monochromator vacuum chamber (Freund *et al.*, 2019 ▸) in the X-ray Tunnel for Distribution XTD1 of the SASE2 beamline, as shown in Fig. 4 ▸. A diamond sensor manipulator was placed about 200 m downstream of the SASE2 undulator and upstream of any beamline optics.

Due to the large signals produced by FEL pulses, each current signal from the diamond detector had to be attenuated by 50 dB using SMA attenuators. The detector was biased with 100 V and the digitization of the signals was performed using commercial ADC modules (Struck company; https://www.struck.de) in a µ-TCA form factor. These ADCs have 16-bit resolution and a sampling rate of up to 125 Megasamples per second (MS s^−1^). Pulse stretchers in the analog front-end electronics prolong the pulses in order to temporally resolve the few-nanoseconds-wide peaks at this limited sampling rate. The ADCs are triggered synchronously with each pulse train.

**Figure 5:**
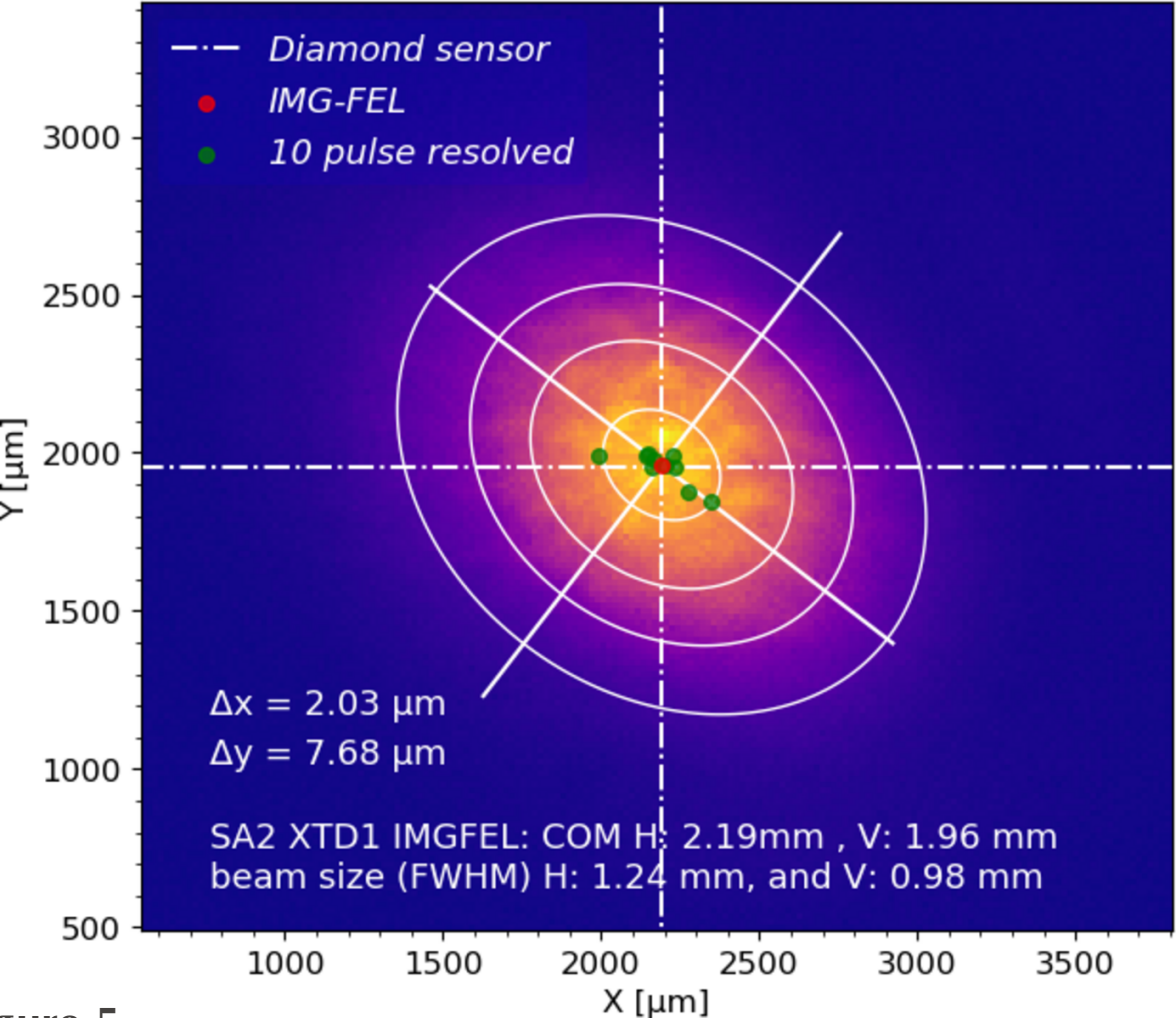
Pulse-resolved position measurement performed with a diamond sensor (ten green dots for ten pulses) at an energy of 7 keV. The red dot is a measurement performed with an FEL imager.

Measurements were performed over several beam times with different photon energies: 12–15 keV and 30 keV. The measurements obtained at a photon energy of 11 keV have been presented by Çonka Yıldız *et al.* (2023 ▸), where the diamond sensor was shown to deliver pulse-resolved beam position information at MHz rate with a measurement uncertainty of 0.5–1% of the beam size. An image of the FEL beam recorded by the FEL imager is shown in Fig. 5 ▸ where the red dot represents the 2D tilted Gaussian fitted centroid position of the beam with an energy of 7 keV. The green dots are single-shot beam positions measured by the diamond sensor at 1.1 MHz rate. The ellipse is the contour plot of the fitted 2D profile, and the dashed cross represents the mean position measured by the diamond sensor.

**Figure 6:**
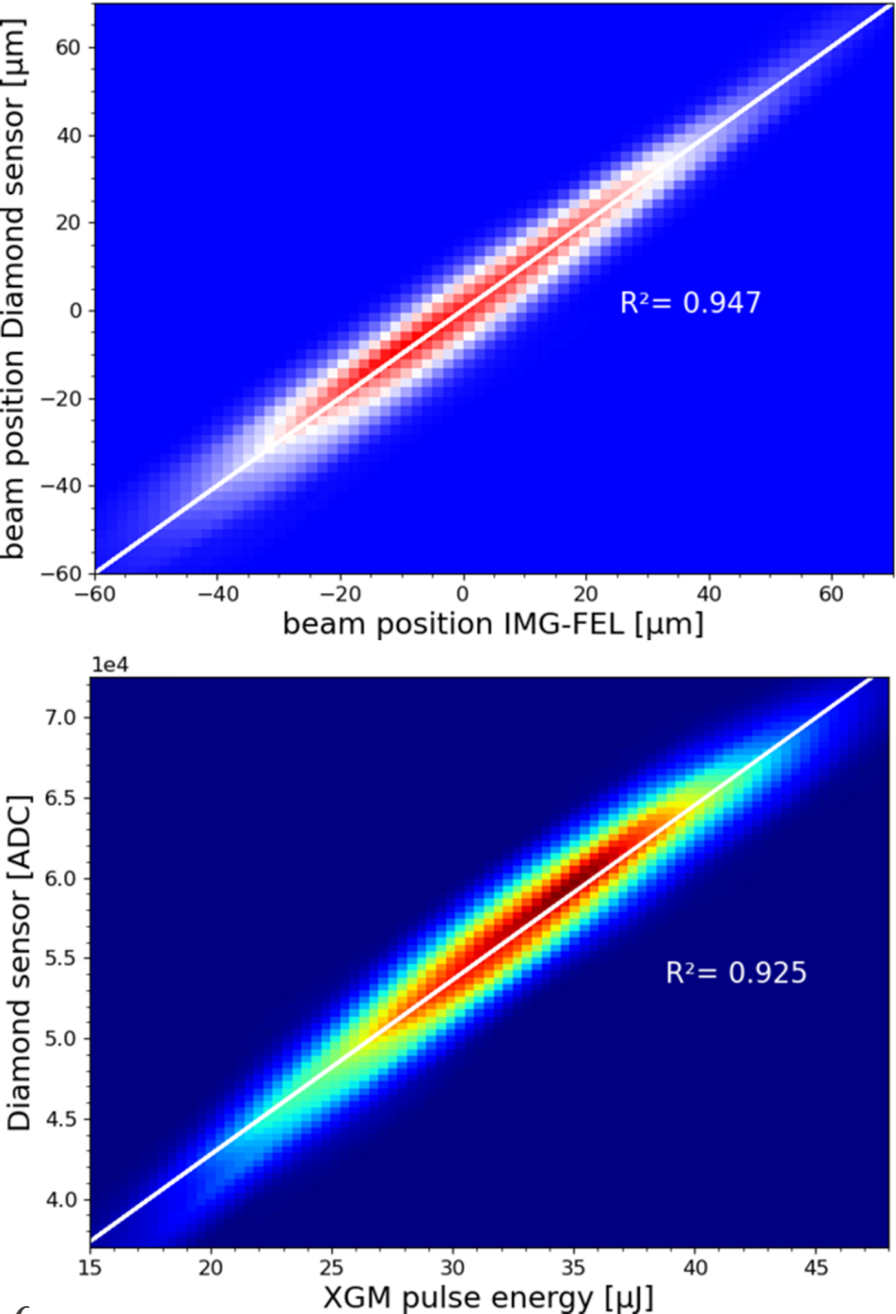
The top plot is the beam position correlation in 2D (IMG-FEL), the bottom plot is the beam energy correlation (XGM). Data were taken with the DLC-coated detector at 27 keV photon energy.

The diamond sensor beam position averaged over two pulses in the train was compared with train-resolved data of the SASE2 FEL imager (Koch *et al.*, 2019 ▸) and demonstrates a good agreement (see upper plot in Fig. 6 ▸). In a similar manner, the pulse energy correlation between the ADC counts of the diamond sensor and the pulse energy from the XGM is shown in the lower plot of Fig. 6 ▸. The 800 µm-wide beam arriving at the diamond had an intra-pulse repetition rate of 2.25 MHz with an average pulse energy of around 35 µJ.

### 3.2. Measurements at the FXE instrument

**Figure 7:**
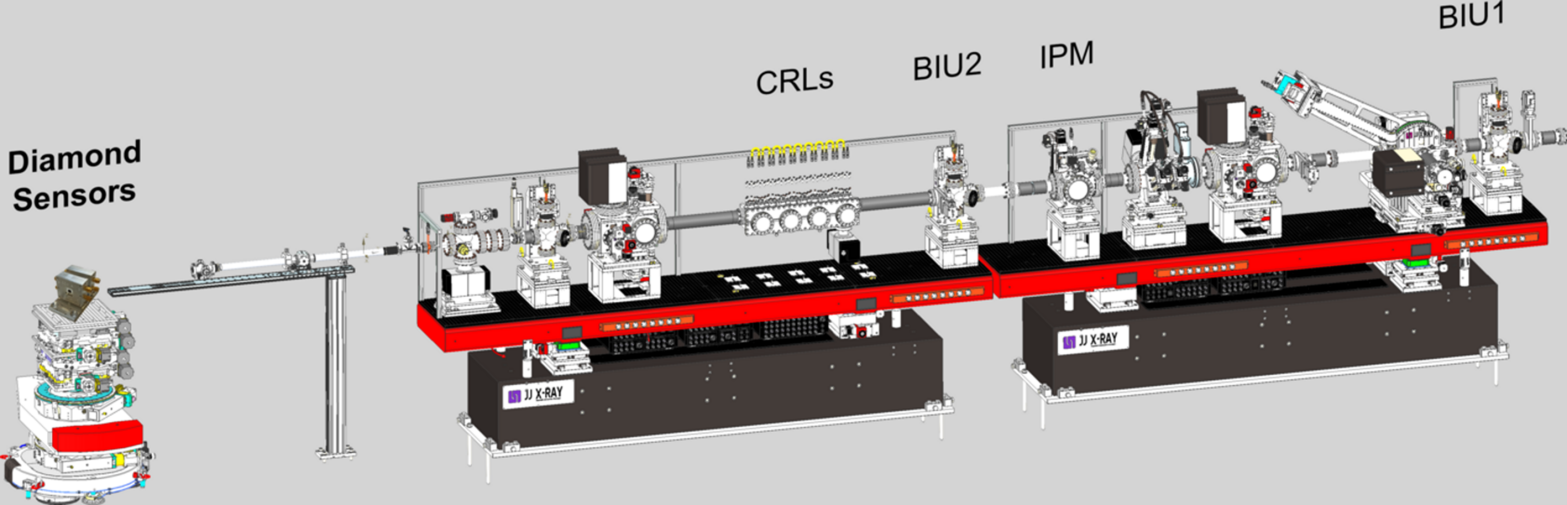
Upstream optics branch of FXE with its various diagnostics and beam shaping components.

The Femtosecond X-ray Experiment (FXE) is an experimental endstation at EuXFEL where the simultaneous application of forward X-ray scattering is combined with simultaneous X-ray spectroscopy techniques at femtosecond time resolution. Further information about the FXE instrument has been given by Galler *et al.* (2019 ▸). An overview of the upstream optics branch of the FXE instrument can be seen in Fig. 7 ▸.

There are various diagnostics components placed upstream and downstream of the measurement position of FXE. Beam imaging units (BIUs) and an intensity monitor (XGM) were used to compare position and energy measurements, respectively, with the diamond sensor data. There is also energy and position data available from an intensity–position monitor (IPM) but not included in this work due to incomplete analysis.

**Figure 8:**
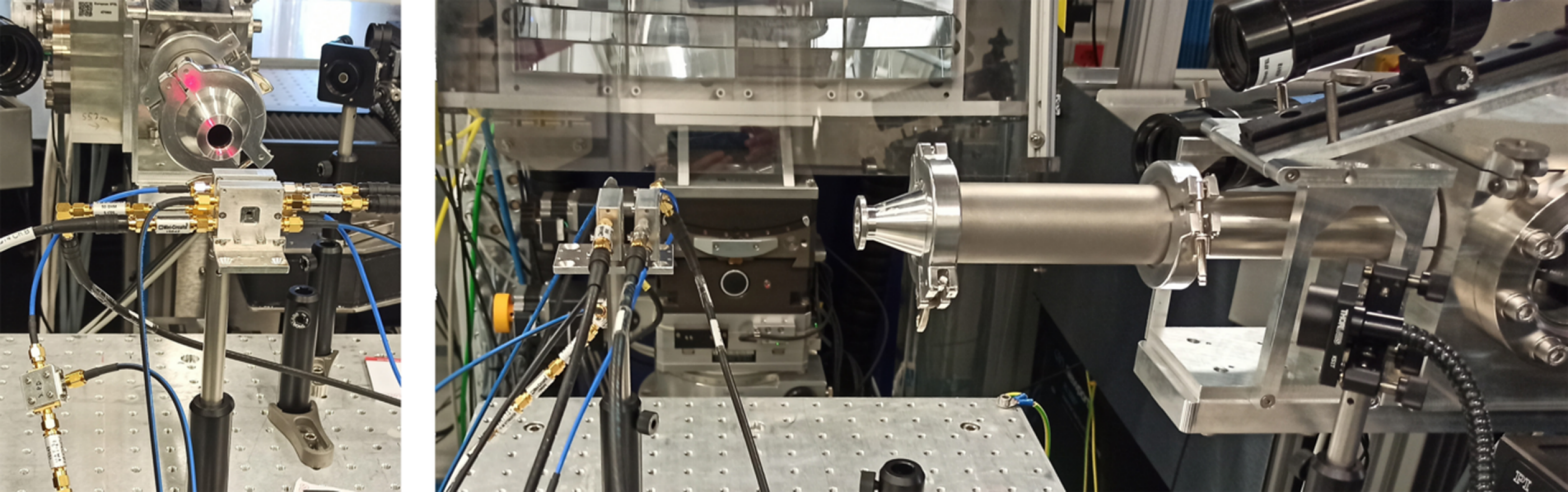
Two different views of the setup at the FXE Hutch to test the new diamond detectors. The X-ray beam propagates to both detectors through the He path exit apperture (round KF flange).

Two recently produced diamond sensors were tested with the X-ray beam for the first time at FXE. They mainly differ in thickness and supplier, namely the company polishing the diamond. The unprocessed diamond material originates in both cases from Element Six. One of the diamonds was polished at ALMAX easyLab bv (Diksmuide, Belgium) while the other was polished at Delaware Diamond Knives (DDK) (Wilmington, Delaware, USA) and was plasma-etched down to the final thickness at CEA (Commissariat à l’Énergie Atomique et aux Énergies Alternatives). The ALMAX diamond sample has a thickness of 40 µm, while the DDK sample is 20 µm thick. They both received an implantation at the University of Augsburg, and the metallization together with further chemical processing was performed at the detector laboratory of the Helmholtzzentrum Gesellschaft für Schwerionenforschung (GSI), Darmstadt. Both sensors were biased with 40 V and had all channels equipped with 20 dB of passive attenuators, whereas the beam itself was not attenuated and was focused down to approximately 0.5 mm. The two diamond sensors were mounted together in air on the same mounting base, so that they could be measured simultaneously to make full use of the X-ray beam time and to cross check the individually measured beam position as shown in Fig. 8 ▸.

**Figure 9:**
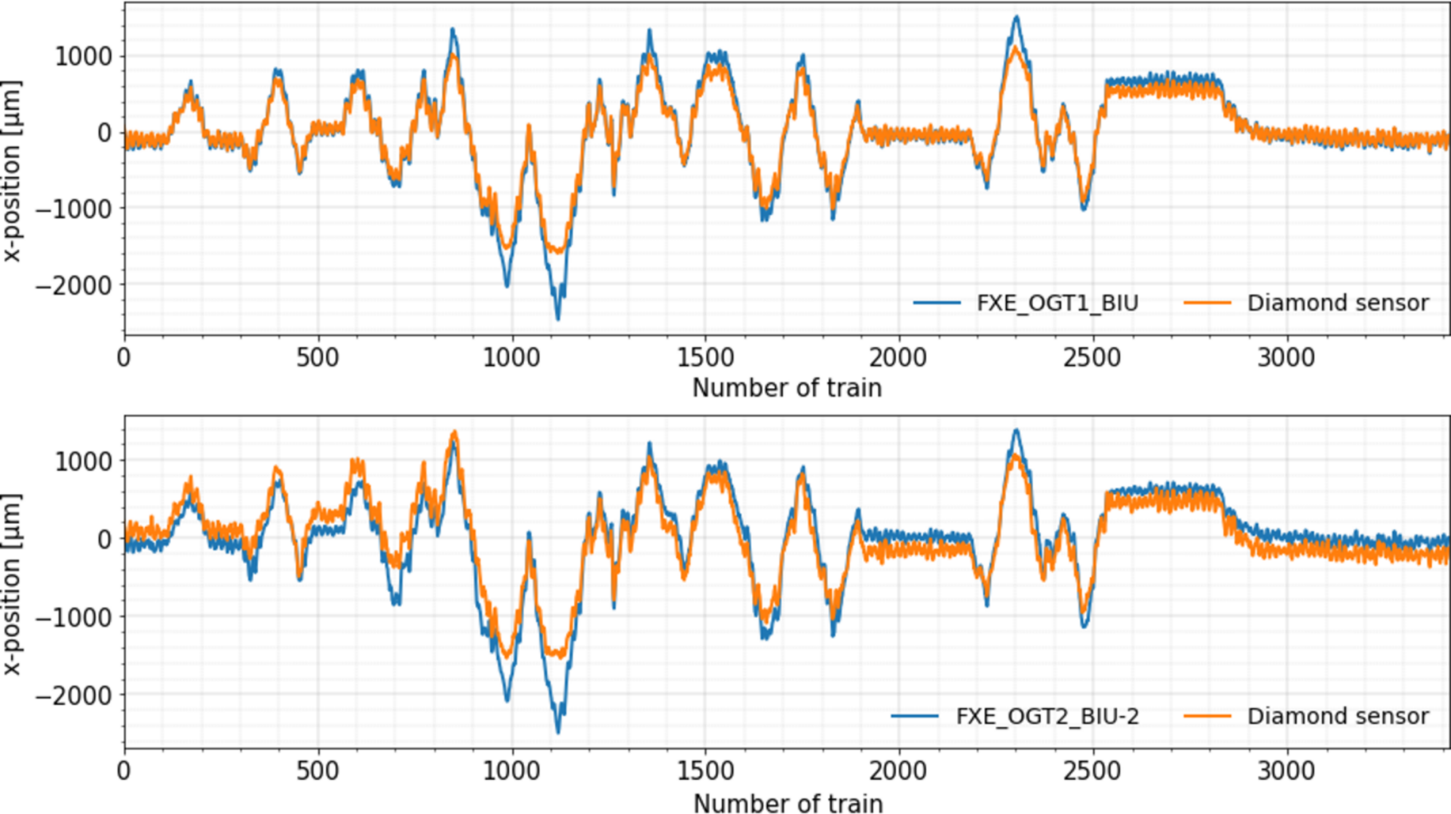
Beam position measured by the DDK detector and BIU (upper plot) and the ALMAX detector and BIU2 (lower plot) in the *x*-direction while the X-ray beam pointing was intentionally randomly varied in the horizontal direction by means of the X-ray mirror angular actuator.

The digitization of the signals is performed by digitizer modules in a µ-TCA crate. Due to the restricted number of available channels, the downstream diamond detector (DDK) was connected to a 14-bit ADC (ADQ14; SP Devices) and the upstream one (ALMAX) to a 12-bit ADC (ADQ412; SP Devices). Several mesh scans of the detectors position in the *x*- and *y*-directions perpendicular to the beam were performed for the coordinate calibration of both detectors. As a result, the beam position information obtained from the diamond sensors were compared with the one simultaneously recorded by the BIUs. Mean position data averaged over 100 pulses at an X-ray energy of 20.5 keV are shown in Fig. 9 ▸ for the horizontal coordinate for the DDK diamond sensor versus BIU and the ALMAX diamond sensor versus BIU2. The beam jitter is larger than usual, as the beam position was intentionally varied by scanning piezo horizontally in a random manner. Beam was neither focused nor attenuated for this run. Both diamond sensors show good agreement with BIUs; the thinner detector matches the track of the BIU slightly better than the thicker one. This may be due to the same bias voltage value applied to them; the thinner detector has better charge collection efficiency, resulting in a better resolution. At the large, slightly truncated peaks, both sensors reach the end of their measuring range when the beam hits the sensor electrode.

**Figure 10:**
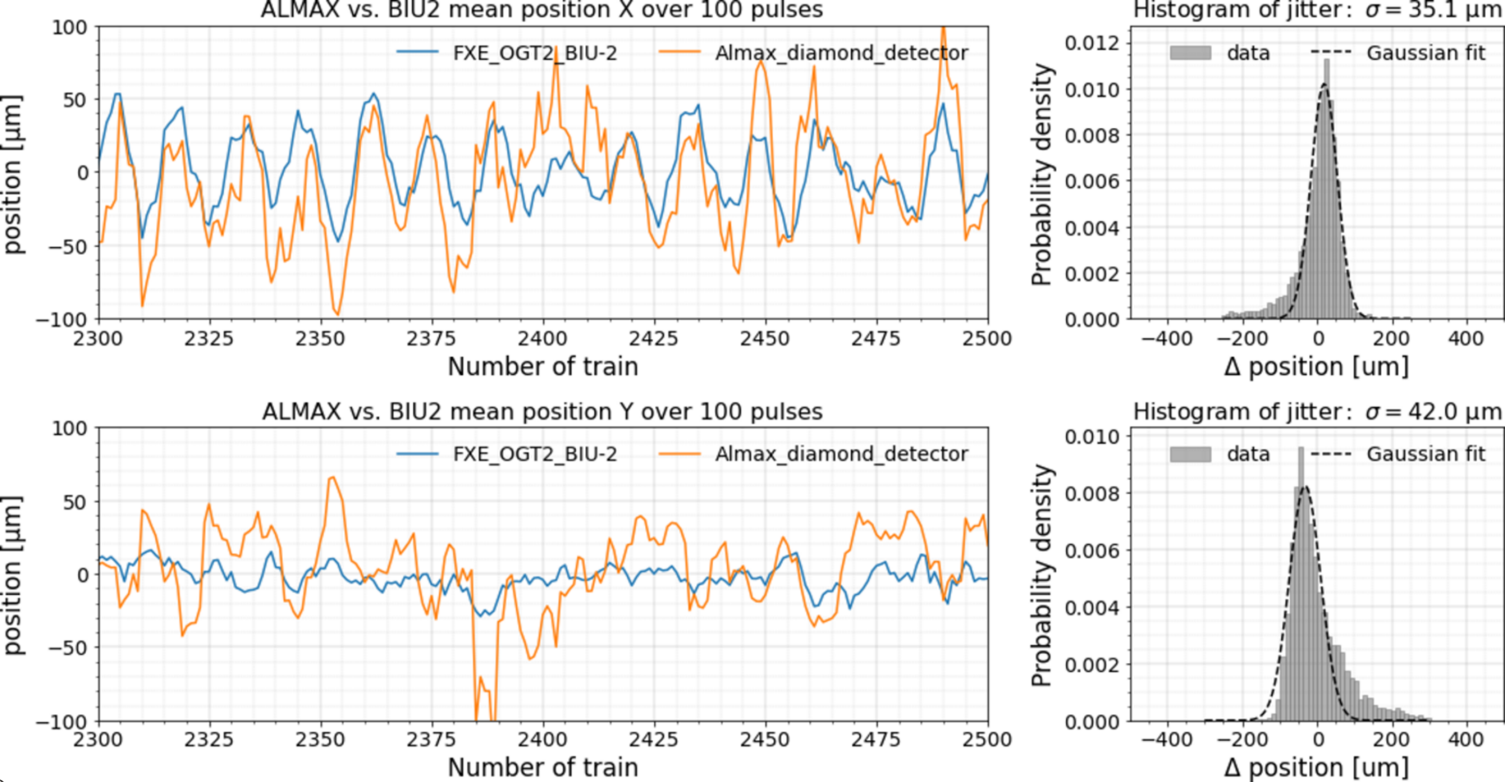
Beam position measured simultaneously in the *x*- and *y*-directions by the ALMAX detector and BIU2 for a focused beam. The histograms on the right show the respective deviation in beam positions determined by BIU2 and ALMAX.

Position measurements of the ALMAX diamond sensor and BIU2 are compared for another run without mirror movement in Fig. 10 ▸. Beam in the previous plot was not focused, whereas in this plot it was focused down to around 500 µm. Here the simultaneous position measurements in both directions are shown for both detectors. The upper part of the figure belongs to the horizontal direction and the lower part is for the vertical direction. The measurements were performed simultaneously for all trains and here only one part is shown to be able to provide a detailed view. There is a better match of the data of the diamond sensor and BIU2 in the horizontal direction, as the BIU demonstrates a lower sensitivity in the vertical direction. In both cases there were 100 pulses in each train and all pulses were used for the mean position calculation and the beam had no attenuation. The histograms located on the right side show the differences between the BIU2 and the diamond detector in the *x*- and *y*-directions for all trains.

Both diamond detectors show a similar performance in comparison with both BIU and BIU2. The position resolution is a bit worse than the measurements performed in the X-ray tunnel. This is mostly due to the reduced biased voltage used for FXE measurements. There are also other factors that are different and may be crucial such as different sensors in terms of thickness and coating, different beam shape, due to distortion from the mirror *etc*.

**Figure 11:**
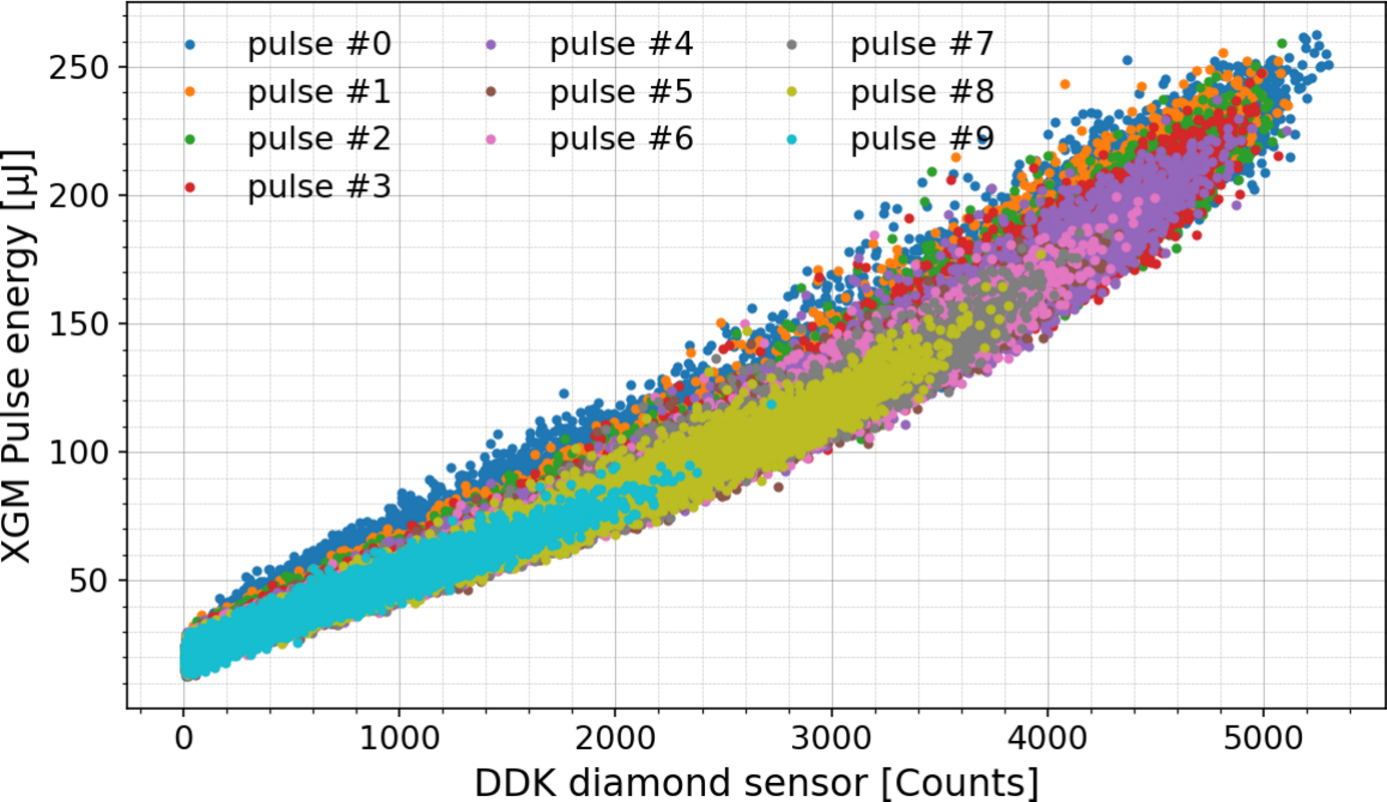
Pulse energy measured by XGM (absolutely calibrated in microjoules) versus DDK detector in ADC counts.

The shot-to-shot pulse energy measurements agree nicely with the XGM data as shown in Fig. 11 ▸. Only the first ten pulses were considered because the rest in the train had a much lower intensity due to machine limitations. These data were taken at a bias voltage of 80 V, since at a lower bias voltage of 40 V some nonlinearity was observed with respect to the XGM. The nonlinear behavior apparent in the plot cannot be attributed to either of the detectors.

**Figure 12:**
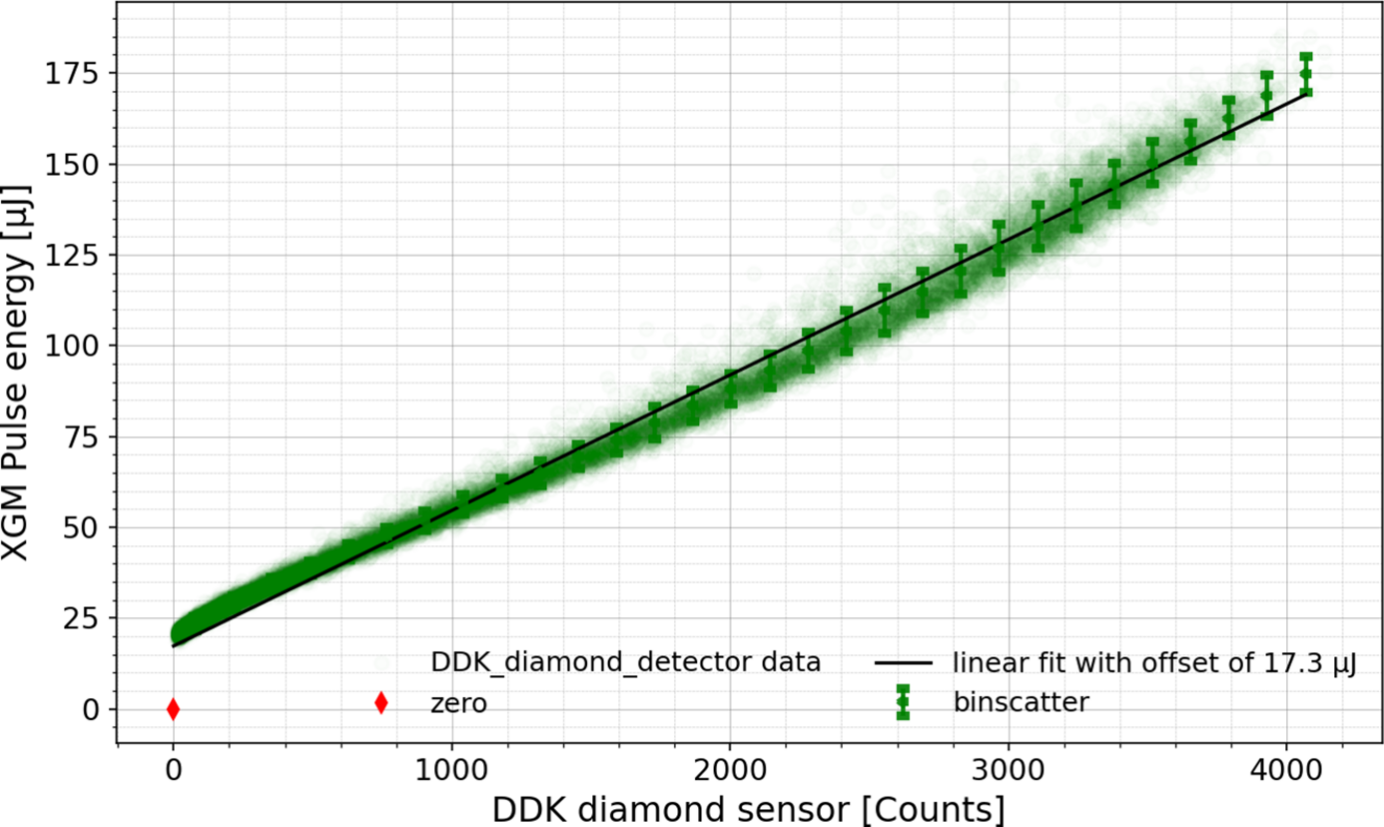
Pulse energy measured by XGM (absolutely calibrated in microjoules) versus DDK detector in ADC counts, averaged over the first ten pulses per train.

Fig. 12 ▸ shows the pulse energy measured by XGM versus DDK energy measurement averaged over the first ten pulses. The correlation plot demonstrates a good degree of linearity with relative maximum deviation of below 10% from the linear regression fit.

## 4. Summary and outlook

Diamond sensors were developed for operation with high-energy X-ray beams such as those produced at FELs. One initial detector was tested under various beam conditions. Two recently produced detectors were tested at the FXE instrument hutch. All measurements show that diamond sensors deliver reliable pulse-resolved beam position and beam energy at a MHz rate. The results are in good agreement with the measurements performed by existing imagers and further devices in the photon tunnel and at the FXE hutch.
